# Identification and field testing of sex-attractant semiochemicals produced by male deer mice, *Peromyscus maniculatus*

**DOI:** 10.1098/rsos.241257

**Published:** 2024-12-18

**Authors:** Elana Varner, Regine Gries, Stephen Takács, Hanna Jackson, Leah Purdey, Daniella Gofredo, Alishba Bibal, Gerhard Gries

**Affiliations:** ^1^ Department of Biological Sciences, Simon Fraser University, Burnaby, British Columbia V5A 1S6, Canada

**Keywords:** volatiles, ketones, testosterone, sex-attractant semiochemicals

## Abstract

Following previous reports that male deer mice, *Peromyscus maniculatus*, produce chemical signals that attract conspecific females, we analysed and field-tested sex-attractant semiochemicals (message-bearing chemicals) of male deer mice. Field traps baited with urine- and faeces-soiled bedding of male mice captured adult female, but not male, mice, indicating dissemination of sex-attractant semiochemicals from the males’ excreta. Analysing excreta headspace volatiles of both males and females by gas chromatography–mass spectrometry revealed that 5-methyl-2-hexanone was male-specific, and that eight other ketones (3-methyl-2-pentanone, 2-hexanone, 4-heptanone, 2-heptanone, 6-methyl-2-heptanone, 3-octanone, 2-octanone, 2-nonanone) were 2.6–5.6 times more abundant in male, than in female, samples. In a field experiment with paired trap boxes, treatment boxes baited with the synthetic ketone lure captured 3.4 times more females (17 : 5) and 1.6 times fewer males (5 : 8) than corresponding unbaited boxes. In a follow-up paired-trap field experiment, treatment boxes baited with both the ketone lure and synthetic testosterone captured 8 times more mature females and 2.3 times more immature females, but 9 times fewer immature males, than control boxes baited only with the ketone lure, all indicating that testosterone is a synergistic sex-attractant semiochemical. As previously shown in house mice, *Mus musculus*, and brown rats, *Rattus norvegicus*, sex-attractant semiochemicals of male deer mice comprise both volatile and sex steroid components.

## 1. Introduction

Macrosomatic rodents use their keen sense of smell during foraging [1,2], for intra- and inter-specific communication [3], and for predator avoidance [4]. Semiochemical-based communication in rodents has been intensely studied, mostly with house mice, *Mus musculus*. In the house mouse system, male-produced semiochemicals (message-bearing chemicals) have both behavioural effects (e.g. attraction of females and deterrence of rival males [5–8]) and physiological effects (e.g. estrus synchronization [9,10] and unfamiliar male-induced spontaneous abortion [11,12]). The semiochemicals disseminate from the males’ urine and faecal deposits. Various compounds contribute to the semiochemical signal or its delivery, such as peptides [9,10], major urinary proteins (MUPs) [11], the sex steroid testosterone [12] and the volatile sex-attractant semiochemicals 2-*sec*-butyl-4,5-dihydrothiazole (thiazole), 7-*exo*-ethyl-5-methyl-6,8-dioxabicyclo[3.2.1]-3-octene (= 3,4-dehydro-*exo*-brevicomin = brevicomin) [13], 1-hexanol and 2,3,5-trithiahexane [14]. Female house mice also produce sex-attractant semiochemicals that attract males [12,15].

Deer mice, *Peromyscus maniculatus*, are pervasive and likely the most common small mammal in North America [16]. They inhabit a wide variety of ecosystems including grasslands, brushy areas, woodlands and forests [17]. Deer mice are considered social rodents tolerating both male and female conspecifics especially during the winter when they may huddle together to conserve heat [18–21]. The basic social unit of deer mice consists of a mature male, a few mature females and several young [19]. The social system may vary in relation to population density, time of year, food availability and other ecological and social factors [22]. Population characteristics change over the course of the year, with high rates of dispersal attributed to the breakdown of established social structures in the spring and autumn [23].

The seasonal reproductive activity of deer mice is linked to photoperiod. When the photophase in the autumn is decreasing, females delay the onset of sexual maturity and males lower the weight of their testes [24]. Males and females mate with multiple partners [25–30], and single litters may have multiple male parentages, indicating the presence of multiple breeding males [25,28]. This type of social or reproductive structure differs from that described for populations of *Apodemus* wood mice [31] and *Mus* house mice [32], where one dominant male is thought to do most of the matings. During the breeding season, female deer mice typically occupy exclusive territories and nest solitarily [22,33] and only females exhibit parental behaviour [34,35] (but see [36]). Deer mice are commonly nocturnal [37] and even limit their foraging activities during full moons to reduce predation risk [38]. The *per capita* foraging activity increased at lower population densities [39].

Deer mice engage in sexual communication, with their semiochemicals causing both physiological and behavioural effects on signal recipients, similar to those described for house mice. Exposure of female deer mice to male semiochemicals may induce spontaneous abortion [40] and estrus-induction [41,42], and urine deposits of adult males attract females and repel rival males [24]. Exposure of juvenile females to male urine accelerates their puberty [43,44] but the urine from castrated males fails to elicit the effect [43], suggesting that semiochemical production is androgen-dependent, as has been shown in house mice [45]. Whereas testosterone is vital for semiochemical production, testosterone itself is a sex-attractant semiochemical of both male house mice and male brown rats, *Rattus norvegicus*, and increases the attraction of females to more volatile sex-attractant semiochemicals [12]. Considering that male deer mice excrete testosterone [46], it follows that testosterone may also be a sex-attractant semiochemical of male deer mice.

To identify semiochemicals of deer mice, Ma *et al*. [47] analysed urine odour profiles of males and females. Many compounds were identified, but no compound was sex-specific or tested for a semiochemical function. However, semiochemicals are not necessarily sex-specific. For example, brevicomin is produced by male house mice (see above) and—together with thiazole—strongly attracts females [48], but brevicomin is also produced by females, albeit at lower quantity [49]. Moreover, semiochemicals may originate from sources other than urine, such as the faeces and facial glands of the signalling sex [15,50].

Working with laboratory-strain deer mice for headspace volatile analyses, and with wild deer mice for field-testing volatiles, we (i) determined whether excreta-soiled bedding of male deer mice attracts wild female deer mice, (ii) identified male-produced volatiles that attract female mice, and (iii) determined whether the male sex steroid testosterone enhances attraction of females to the volatile blend. We refer to ‘semiochemicals’ rather than ‘pheromone components’ in deer mice, because the expanded operational definition of pheromone requires not only evidence that a chemical, or a blend of chemicals, is released by a signaller and prompts a behavioural response by conspecific signal recipients—as originally defined [51]—but also that *all* blend constituents are ‘necessary and sufficient to elicit the full response’ [52]. Whereas functional roles of all blend constituents are readily investigated with abundant insect populations in consecutive field experiments, equivalent data in field experiments with mammals are exceedingly time-consuming to collect, particularly when the chemical blend is highly complex, like in our study.

## 2. Material and methods

### 2.1. Laboratory animals

Sexually mature deer mice, *P. maniculatus bairdii*, were obtained in two separate shipments from the Peromyscus Genetic Stock Center (University of South Carolina, Columbia, SC, USA) and housed in Animal Care Services (ACS) of Simon Fraser University (SFU). The first cohort of mice (females and males) arrived in March 2019 and was used for the identification of semiochemicals, which was accomplished within the following six weeks. The second cohort of mice (males only) arrived in September 2023 and was used within the following seven weeks to produce bedding soiled with male excreta for testing its effect on attraction of wild females.

Upon arrival, females (which are tolerant of each other) were housed in three groups of five (each cage: 45 × 23 × 15 cm^3^), whereas each of 15 males in the first shipment and each of six males in the second shipment were housed singly to avoid potential aggression among males (each cage: 20 × 37 × 14 cm^3^). Cages of females (5–6 months old) and males (7–8 months old) were lined with 450 and 150 g, respectively, of corncob bedding (Anderson’s Bed o’cobs, The Andersons Inc., Maumee, OH, USA) and fitted with a single Nalgene dome (Jaimeson’s Pet Food Distributers, Richmond, BC, Canada). Urine- and faeces-soiled bedding was replaced with fresh bedding every 2 weeks. Rodent food (LabDiet^®^ Certified Rodent Diet, LabDiet, St Louis, MO, USA) and water were provided ad libitum. Mice were kept on a reversed photoperiod of 12L : 12D in rooms maintained at about 50% relative humidity and 21°C. The research protocol was approved and supported by the Animal Care Committee of SFU (protocol 1295B-19), which abides by the Canadian Council on Animal Care guidelines.

### 2.2. Chemicals

All chemicals (% purity) were purchased from Sigma-Aldrich: 3-methyl-2-pentanone (99%), 2-hexanone (98%), 5-methyl-2-hexanone (99%), 4-heptanone (98%), 2-heptanone (99%), 6-methyl-2-heptanone (>95%), 3-octanone (98%), 2-octanone (98%), 2-nonanone (99%).

### 2.3. Does excreta-soiled bedding of male deer mice attract wild female deer mice?

**Figure 1.**
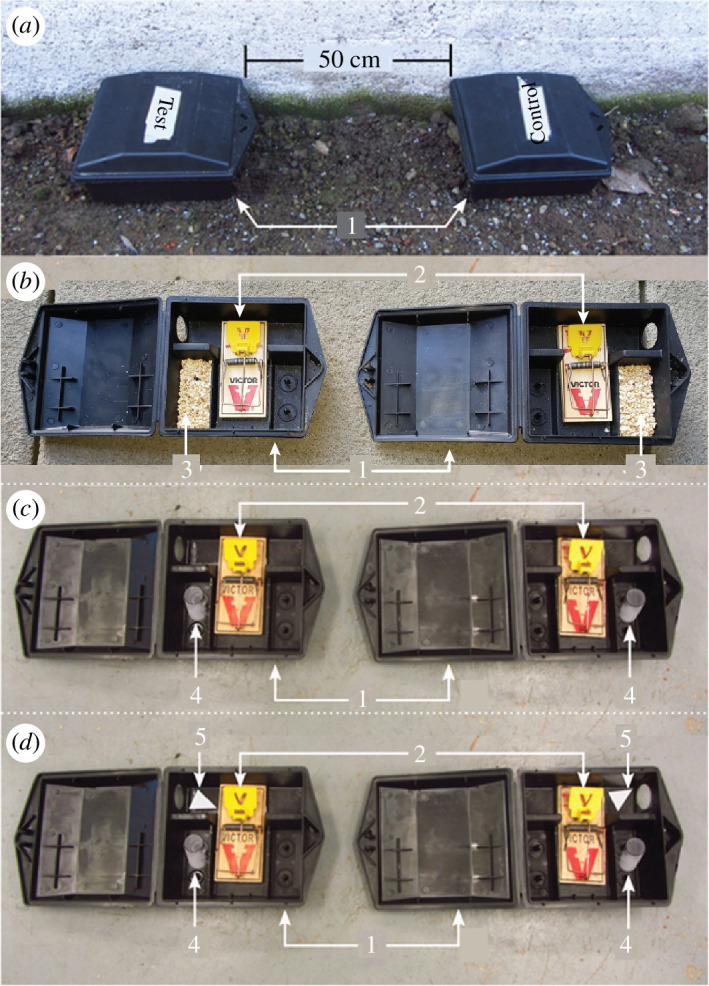
Photographs illustrating (*a*) the paired-trap experimental design deployed in field experiments, and (*b–d*) detailed views of the food bait and semiochemical lure tested in randomly assigned treatment and control traps, as follows: **1** = trap box, **2** = snap trap with food bait [53], **3** = bedding soiled with urine and faeces excreta of male deer mice or clean bedding (control); **4** = glass scintillation vial (20 ml) containing a blend of candidate sex-attractant semiochemicals formulated in mineral oil (10 ml) or mineral oil alone (10 ml; control); and **5** = piece of filter paper treated either with testosterone (750 ng) dissolved in acetonitrile (50 μl) or with acetonitrile alone (50 μl; control).

To determine whether urine and faeces excreta in soiled bedding of male deer mice release semiochemicals that attract wild female deer mice, a field experiment (Exp. 1) was run on three private premises (Sunshine Valley, British Columbia; Canada; 49.274953, −121.236601 to 49.273920, −121.233998) between 16 October and 1 November 2023. All premises in Exp. 1 and in experiments 2–3 (see below) were deer mouse-inhabited based on occasional visual observations of deer mice but the population density was not specifically assessed. Seventeen pairs of trap boxes were deployed concurrently (PROTECTA^®^ Mouse, Bell Laboratories Inc. Madison, WI, USA), with 0.5 m spacing between the boxes in each pair (figure 1*a*), and at least 2 m between pairs. Trap box pairs were placed along the interior and exterior walls of buildings and wood piles. Both the treatment and the control trap box in each trap box pair were fitted with a Victor^®^ snap trap (M325 M7 Pro mouse Woodstream Co., Lititz, PA, USA) baited with peanut butter which prompted feeding and thereby capture of responding mice. The randomly assigned treatment trap in each pair was baited with bedding (30 g) soiled by male deer mice, whereas the corresponding control trap received clean bedding (30 g) (table 1). Soiled bedding was collected daily from the heavily soiled ‘latrine’ cage corners of six singly housed male deer mice and was stored at room temperature in Ziploc bags (retaining odour) for no more than 48 h before it was placed into treatment traps. Every day, traps were checked, the bedding was replaced, and the position of trap boxes within each pair was randomized. In addition, once each week, the snap traps, trap boxes and food baits were replaced. Whenever a mouse had been captured, its sex and maturity were recorded, a new trap box and snap trap were deployed in the same location, but the position of the treatment and the control box within a trap box pair was re-randomized. Sex and sexual maturity of the captured mouse were determined based on ano-genital distance [54] and genitalia development, such as visibly discernible testes of sexually mature males [55] and vaginal opening of sexually mature females [56], bearing in mind that the ano-genital distance can vary between specimens, that testis size may change through the season [24], and that juveniles may be present year-round under favourable conditions [21].

**Table 1.** List of stimuli tested in paired-trap field experiments 1−3; *n* = number of trap box pairs with deer mouse captures.

treatment trap box	control trap box
*Exp. 1 (n = 19)* ^a^ *: Does bedding soiled with urine and faeces excreta of male deer mice attract wild female deer mice?*	
— bedding soiled with male deer mouse excreta^b^ — peanut butter food bait	— clean bedding — peanut butter food bait
*Exp. 2 (n = 36)*^c^*: Do synthetic CSSs*^d^ *identified in male deer mice excreta attract wild female deer mice?*	
— volatile CSSs^e^^,f^ in 10 ml mineral oil — grain-based food bait^g^	— mineral oil (10 ml) — grain-based food bait
*Exp. 3 (n = 40)* ^h^ *: Does testosterone enhance the attractiveness of CSSs to wild female deer mice?*	
— volatile CSSs^e^^,f^ in 10 ml mineral oil + testosterone on filter paper^i^ — peanut butter food bait	— volatile CSSs^e^^,f^ in 10 ml mineral oil — peanut butter food bait

^a^ There were 17 concurrently run trap-box pairs. In two of these pairs, a mouse was captured twice, resulting in a total of 19 replicates (after the first capture, a new trap box and snap trap were deployed in the same location, but the position of the treatment and the control box was re-randomized).

^b^ Corncob bedding (30 g) collected in 24 h intervals from the heavily soiled ‘latrine’ cage corners of 6 singly housed male deer mice.

^c^ There were 25 concurrently run trap-box pairs, of which 10 captured a mouse twice, resulting in a total of 35 replicates (see also footnote a).

^d^ CSSs: candidate sex-attractant semiochemicals.

^e^ CSS blend: 3-methyl-2-pentanone (0.03 mg), 2-hexanone (0.01 mg), 5-methyl-2-hexanone (0.02 mg), 4-heptanone (0.03 mg), 2-heptanone (0.8 mg), 6-methyl-2-heptanone (0.03 mg), 3-octanone (2.1 mg), 2-octanone (0.3 mg), 2-nonanone (0.7 mg) formulated in mineral oil (10 ml).

^f^ The synthetic ketones were formulated in milligram amounts, in mineral oil, to achieve their emission at *nanogram* amounts comparable to those from the excreta of deer mouse males over the same time period (figure 3*a*,*c*).

^g^ Composition reported in Takács *et al*. [53].

^h^ There were 30 concurrently run trap-box pairs, of which 10 captured a mouse twice, resulting in a total of 40 replicates (see also footnote a).

^i^ Testosterone (750 ng) was dissolved in acetonitrile (50 μl) and applied to filter paper, whereas the filter paper in the control trap was likewise treated with 50 μl of acetonitrile. Testosterone was dispensed from filter paper because the steroid has significantly different release characteristics from the ketones formulated in mineral oil. The amount of testosterone tested represents 1.5 times the amount of testosterone present in 1 g of male deer mouse faeces [46].

### 2.4. Identification of male-produced volatile semiochemicals that attract females

#### 2.4.1. Collection of urine and faeces headspace volatiles from female and male deer mice

**Figure 2.**
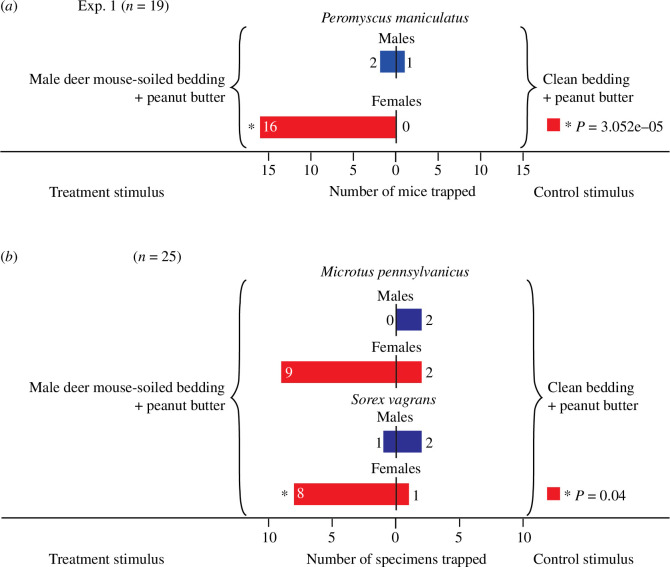
Captures of (*a*) mature deer mice, *Peromyscus maniculatus*, and (*b*) eastern meadow voles, *Microtus pennsylvanicus*, and vagrant shrews, *Sorex vagransii*, in paired traps in Exp. 1. The randomly assigned treatment trap in each pair was baited with bedding soiled with urine and faeces of male deer mice, whereas the corresponding control trap received clean bedding. The asterisk (*) denotes significantly more captures of females in traps baited with soiled bedding than in traps fitted with clean bedding (binominal tests: female deer mice: *p* = 3.052 × 10^−5^; female shrews: *p* = 0.04).

With our field data showing that traps baited with excreta-soiled bedding of male deer mice attract and capture wild female deer mice (see §3; figure 2), we proceeded to identify the volatiles that mediated the attraction. For each experimental replicate (*n* = 5 for both female and male mice), excreta-soiled bedding (see above) was collected separately from five group-housed females (450 g bedding) disregarding their estrus cycle, and from three singly housed males (3 × 150 g = 450 g of total bedding). The bedding was placed into separate Pyrex glass chambers (30 × 15 cm^2^) connected to a Pyrex glass tube (15 cm × 5 mm OD) filled with the adsorbent Porapak Q (200 mg) which served as a volatile trap. Charcoal-filtered air was drawn at a flow of 1 l min^−1^ through each chamber and the Porapak Q volatile trap. After capturing urine and faeces volatiles on Porapak Q for 24 h, volatiles were desorbed with consecutive rinses of pentane (2 ml) and ether (2 ml), dodecyl acetate was added as an internal standard, and extracts were concentrated under a fine stream of nitrogen to 250 µl per sample.

#### 2.4.2. Identification and quantification of urine and faeces headspace volatiles from male and female deer mice

Aliquots (2 µl) of Porapak Q extracts were analysed on a Varian Saturn Ion Trap GC-MS fitted with a DB-5 MS GC column (30 m × 0.25 mm internal diameter, film thickness 0.25 µm; Agilent Technologies Inc., Santa Clara, CA, USA) using helium as the carrier gas (35 cm s^−1^), and running the following temperature programme: 40°C (5 min), 10°C min^−1^ until 280°C (5 min). The injector port was set at 250°C and the ion trap at 200°C. Volatiles were identified by comparing their retention indices (relative to straight-chain alkanes [57]) and mass spectra with those of authentic standards. Volatiles that were either male-specific (5-methyl-2-hexanone), or that were more abundant, albeit not statistically, in headspace volatiles of males than females (2-hexanone, 3-octanone, 2-heptanone, 2-nonanone, 2-octanone, 6-methyl-2-heptanone, 3-methyl-2-pentanone, 4-heptanone) (see §3) were considered candidate sex-attractant semiochemicals (CSSs). In total ion chromatograms, the amount of each ketone was quantified by comparing its area count with that of an internal standard (dodecyl acetate). These amounts were then divided by the number of mice in the sample (5 females or 3 males) to obtain the ketone amounts produced by a single deer mouse.

#### 2.4.3. Effect of a candidate sex-attractant semiochemical trap lure on captures of deer mice in field settings

This field experiment (Exp. 2) was run in three deer mouse-inhabited premises in the Greater Vancouver and Abbotsford areas of British Columbia, Canada, between September 2019 and April 2021. The experimental design was identical to that as described above except that soiled bedding was replaced with a synthetic trap box lure, and that peanut butter as the snap trap bait (see Exp. 1) was replaced with a bait containing attractive food odourants and grain-based feeding stimulants [53]. Using a different type of food bait in Exp. 2 (and in Exp. 3, see below) was a measure to help ensure that captures in experiments were not driven by the type of food bait deployed.

**Figure 3.**
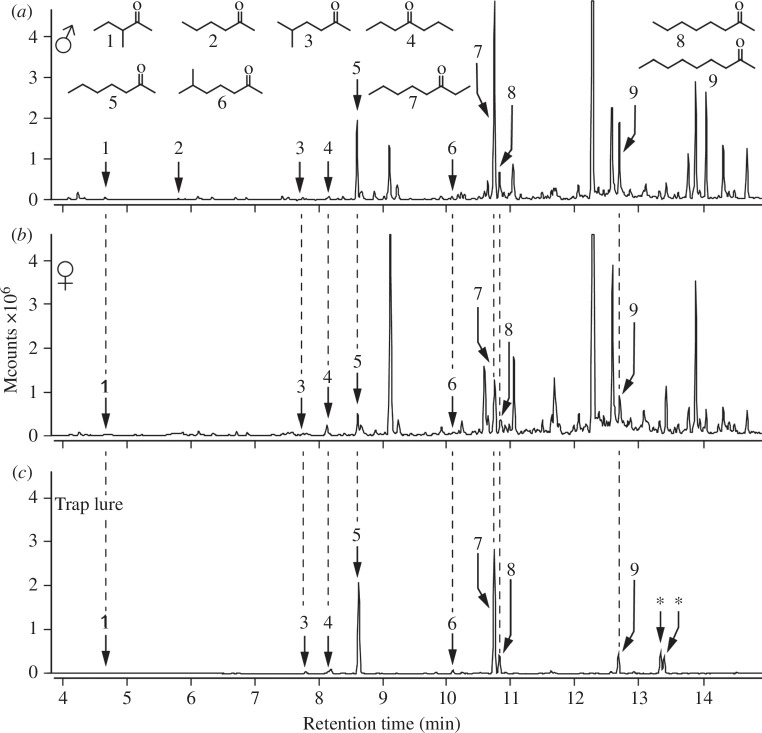
Total ion chromatograms of headspace volatile extracts obtained from (*a*) bedding soiled with urine and faeces excreta from three male deer mice, (*b*) bedding soiled with excreta from five female deer mice and (*c*) a trap lure mineral oil formulation of synthetic ketones (5 days after preparation) that was tested in field experiments with wild deer mice (see §2 for details). Note: (1) 5-methyl-2-hexanone (**3**) was male-specific, and eight other ketones (3-methyl-2-pentanone (**1**), 2-hexanone (**2**), 4-heptanone (**4**), 2-heptanone (**5**), 6-methyl-2-heptanone (**6**), 3-octanone (**7**), 2-octanone (**8**), 2-nonanone (**9**)) were 2.6–5.6 times more abundant, on average, in samples of three males each (*n* = 5) than in samples of five females each (*n* = 5) (see table 1); (2) 2-hexanone (**2**) was not present in this particular female sample but was detectable in other samples; (3) in (*c*), the two compounds marked by an asterisk are contaminants; (4) the headspace profile of synthetic ketones emanating from the trap lure during 24 h greatly resembles that of natural ketones emanating from soiled bedding of three deer mouse males during 24 h; (5) mass spectrometric analysis: Varian Saturn Ion Trap GC-MS; DB-5 MS GC column; temperature programme: 40°C (5 min), 10°C min^−1^ to 280°C (5 min).

Traps within each of 25 concurrently deployed trap-box pairs received a 20 ml glass scintillation vial containing either a 4 mg CSS blend (3-methyl-2-pentanone (0.03 mg), 2-hexanone (0.01 mg), 5-methyl-2-hexanone (0.02 mg), 4-heptanone (0.03 mg), 2-heptanone (0.8 mg), 6-methyl-2-heptanone (0.03 mg), 3-octanone (2.1 mg), 2-octanone (0.3 mg), 2-nonanone (0.7 mg)) formulated in mineral oil (10 ml) or an unscented mineral oil control (10 ml) (figure 1*b*; table 1). To determine whether the CSS trap lure released these ketones at biologically relevant amounts, headspace ketones from the mineral oil formulation (trap lure) were captured on Porapak Q for 24 h and were analysed and quantified as described for natural ketones. The synthetic ketones were formulated in milligram amounts, in mineral oil, to achieve their emission at *nanogram* amounts comparable to those from the excreta of deer mouse males over the same time period (figure 3*a*,*c*). More specifically, on the day the lure was formulated, its ketone release rate was only three times higher than that of the top excreta release rates, and after ageing 5 days the lure’s ketone release rate had declined by 30%. These data, coupled with the fact that trap lures were replaced in 7-day intervals (see below), indicate that the ketone release rate of the field-tested lure was well within biological relevance.

Traps were checked twice each week and the food bait and ketone lure were replaced once each week. When a mouse had been captured, its sex and maturity were recorded, the trap box and snap trap were replaced, and the position of the treatment and the control box within a trap-box pair was re-randomized.

### 2.5. Does the male sex steroid testosterone enhance attraction of females to volatile semiochemicals

**Figure 4.**
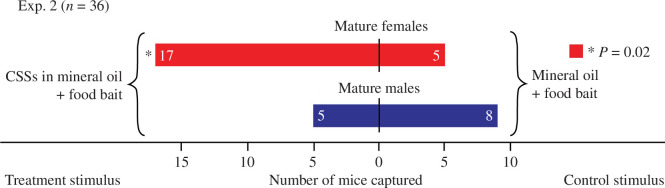
Captures of mature female and male deer mice, *Peromyscus maniculatus*, in paired traps (figure 1*a*) in Exp. 2. Both boxes in each pair received a glass scintillation vial (20 ml; figure 1*b*) containing either a 4 mg blend of the candidate sex-attractant semiochemicals (CSSs) (3-methyl-2-pentanone (0.03 mg), 2-hexanone (0.01 mg), 5-methyl-2-hexanone (0.02 mg), 4-heptanone (0.03 mg), 2-heptanone (0.8 mg), 6-methyl-2-heptanone (0.03 mg), 3-octanone (2.1 mg), 2-octanone (0.3 mg), 2-nonanone (0.7 mg)) formulated in mineral oil (10 ml) or a mineral oil (10 ml) control stimulus. The asterisk denotes significantly more captures of females in traps baited with the CSS lure (binomial test, *p* = 0.02).

With field data showing that traps baited with the CSS lure attract and capture wild female deer mice (see §3; figure 4), we proceeded to Exp. 3, testing the ability of testosterone to enhance captures of female mice in CSS-baited traps. Exp. 3 was run between 1 November 2021 and 17 January 2022 on two private premises (Sunshine Valley) and two commercial premises (Greater Vancouver area, British Columbia, Canada) inhabited with deer mice. Each of 30 concurrently deployed trap-box pairs was set up and serviced as described for Exp. 2. Both trap boxes in each pair were baited with the CSS lure (see Exp. 2), and the treatment box also received a piece of filter paper (Whatman No. 1, 120 mm, Maidstone, UK) to which testosterone (750 ng) dissolved in acetonitrile (50 μl) was applied (table 1). This dose represents 1.5 times the amount of testosterone present in 1 g of male deer mouse faeces [46]. The filter paper in the control trap was likewise treated with 50 μl of acetonitrile. Testosterone was dispensed from filter paper because the steroid has significantly different release characteristics from those of the ketones formulated in mineral oil in the CSS lure.

### 2.6. Data analyses

Capture data of female deer mice, and by-capture data of female voles and shrews, in treatment and control traps of field experiments 1−3 were analysed by a binomial test. The quantities of each ketone in five samples of males and five samples of females were compared using unpaired 2-tailed *t*-tests. Data were analysed using R (version: 4.4.1) and R Studio (version: 2024.04.2+764) [58]. Residual normality and homoscedasticity were assessed using diagnostic plots. All test assumptions were met. We have also uploaded an R studio project folder. Capture data of male mice, voles and shrews in Exp. 1, as well as of mature male mice in Exp. 3, were all deemed too low to warrant reporting of statistical analyses.

## 3. Results

### 3.1. Does excreta-soiled bedding of male deer mice attract wild female deer mice?

Bedding soiled by male deer mice affected trap captures of wild female—but not wild male—deer mice. Traps baited with soiled bedding captured 16 adult female deer mice, whereas traps fitted with clean bedding captured none (Exp. 1: *p* = 3.052 × 10^−5^, 95% confidence interval (95% CI) = 0.79−1.00; figure 2*a*), indicating strong attraction of females to sex-attractant semiochemicals disseminating from the urine or faeces excreta of conspecific males. Conversely, traps baited with soiled and clean bedding captured 2 and 1 adult male deer mice, respectively (figure 2*a*), suggesting that male deer mice are not attracted to semiochemical signals of conspecific males.

Interestingly, traps baited with soiled bedding also attracted females of eastern meadow voles, *Microtus pennsylvanicus* (treatment to control captures: 9:2; *p* = 0.07, 95% CI = 0.48–0.98; figure 2*b*), and vagrant shrews, *Sorex vagrans* (8:1; *p* = 0.04, 95% CI = 0.52–0.10; figure 2*b*), suggesting that males of deer mice, eastern meadow voles, and vagrant shrews share one or more semiochemicals.

Furthermore, traps baited with soiled bedding captured one female each of the bushy-tailed woodrat (packrat), *Neotoma cinerea*, and the Douglas squirrel, *Tamiasciurus douglasii.*

### 3.2. Identification of male deer mouse volatile semiochemicals that attract females

#### 3.2.1. Identification of excreta headspace volatiles from male and female deer mice

Comparative GC-MS analyses of the headspace volatiles emanating from urine- and faeces-soiled bedding of adult male and adult female deer mice revealed various groups of organic compounds including ketones, acids and alcohols. Among nine ketones, one ketone (5-methyl-2-hexanone) was male-specific, and eight (2-hexanone, 3-methyl-2-pentanone, 4-heptanone, 2-heptanone, 6-methyl-2-heptanone, 3-octanone, 2-octanone, 2-nonanone) were 2.6–5.6 times more abundant in samples of males than in samples of females (figure 3*a*,*b*; table 2). All of these nine ketones were deemed CSSs.

**Table 2.** Quantitative comparison of nine ketones in headspace volatiles of urine and faeces excreta from single male and female deer mice.

	mean (± s.e.) and [top] amounts (ng)^a^ of ketones in headspace volatile extract			
compounds	males	females	male–female differential	*p*-value^b^
3-methyl-2-pentanone	57.58 (7.155) [63]	10.33 (5.42)	5.6	0.002
2-hexanone	21.38 (10.73) [20]	7.36 (4.53)	2.9	0.26
5-methyl-2-hexanone	9.50 (9.167) [46]	0.00 (0)	N/A	0.35
4-heptanone	21.68 (11.74) [63]	7.47 (5.03)	2.9	0.30
2-heptanone	550.36 (251.82) [1536]	111.13 (55.05)	5.0	0.13
6-methyl-2-heptanone	17.38 (12.62) [67]	4.44 (3.70)	3.9	0.36
3-octanone	1235.18 (775.71) [4294]	234.92 (163.35)	5.35	0.24
2-octanone	153.52 (102.71) [553]	59.47 (53.86)	2.6	0.44
2-nonanone	404.87 (247.66) [1371]	80.46 (59.69)	5.0	0.24
total ketones	1976.45 [8013]	515.58	3.8	

^a^ Ketones were present in headspace volatiles of bedding (450 g) soiled with faeces and urine from three laboratory-kept male deer mice or five female deer mice during 2 weeks. Headspace volatiles of such soiled bedding were collected over 24 h and quantified in total ion chromatogram analyses (figure 3). The amount of each ketone was derived by comparing its area count with that of an internal standard (dodecyl acetate). These amounts were then divided by the number of mice in the sample (5 females or 3 males) to obtain the ketone amounts produced by a single mouse. There were 5 samples each of males and females.

^b^ The quantities of each ketone in 5 samples of males and in 5 samples of females were compared by unpaired 2-tailed *t*-tests.

#### 3.2.2. Effect of a candidate sex-attractant semiochemical trap lure on captures of deer mice in field settings

Synthetic CSS trap lures—with a headspace volatile profile resembling that of male deer mouse soiled bedding (figure 3*a*,*c*)—increased field captures of female deer mice. CSS-baited traps captured 17 mature female deer mice, whereas unbaited paired control traps captured only five females (Exp. 2: *p* = 0.02, 95% CI = 0.55–0.92; figure 4). Conversely, CSS-baited traps and unbaited paired control traps captured five and eight mature male deer mice, respectively (Exp. 2: *p* = 0.58, 95% CI = 0.14–0.68; figure 4). There were no captures of immature male or female deer mice, or any other animals, in this experiment.

### 3.3. Does testosterone enhance attraction of females to volatile sex-attractant semiochemicals?

**Figure 5.**
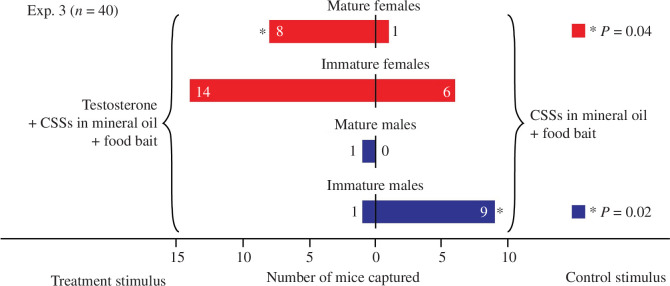
Captures of mature and immature female and male deer mice, *Peromyscus maniculatus*, in paired traps (figure 1*a*) in Exp. 3. Both boxes in each pair received the blend of volatile candidate sex-attractant semiochemicals (CSSs) formulated in mineral oil (see caption of figure 4). The treatment box in each pair also received a piece of filter paper (figure 1*c*) treated with testosterone (750 ng) dissolved in acetonitrile (50 μl), whereas the filter paper in the corresponding control trap received acetonitrile only (50 μl). The asterisks denote significantly more captures of mature females (binomial test, *p* = 0.04), and fewer captures of immature males (binomial test, *p* = 0.02), in testosterone-baited traps. Note that the amount of testosterone represents 1.5 times the amount of testosterone present in 1 g of male deer mouse faeces [46].

Testosterone increased captures of female deer mice in CSS-baited traps. Traps baited with both the CSS lure and testosterone captured eight mature and 14 immature female deer mice, whereas traps baited only with the CSS lure captured one mature and six immature female deer mice (Exp. 3: mature females: *p* = 0.04, 95% CI = 0.52–0.10; immature females: *p* = 0.12, 95% CI = 0.46–0.88; figure 5). Conversely, traps baited with both the CSS lure and testosterone captured one mature and one immature male deer mouse, whereas traps baited only with the CSS lure captured nine immature male deer mice (Exp. 3: immature males: *p* = 0.02, 95%, CI = 0.002−0.45; figure 5). Capture of only one mature male mouse in this experiment did not warrant statistical analysis. No rodents other than deer mice were captured in Exp. 3.

## 4. Discussion

Our study demonstrates that excreta-soiled bedding of male deer mice does attract wild female deer mice. Analysing the headspace volatiles of this bedding revealed nine ketones as CSSs. A synthetic CSS lure also attracted wild female deer mice, and the male sex steroid testosterone enhanced attraction of females to the CSS lure.

As sex-attractant semiochemicals may originate from either urine or faeces deposits of the signalling sex, as shown with house mouse females [15], we took an inclusive approach and collected headspace volatiles from bedding soiled with both urine and faeces of male deer mice. Moreover, while previous studies have used frozen urine for subsequent analysis [47], we captured headspace volatiles exclusively from urine and faeces excreta that were never subjected to freezing given that temporary freezing of urine may alter signal characteristics, as shown with house mice [59]. Finally, as sex-attractant semiochemicals are typically deemed sex-specific, or at least more abundant in the signalling sex, we compared urine/faeces headspace volatiles from males and females.

As expected, based on a previous study [47], the urine/faeces headspace volatiles of male and female deer mice were complex, comprising many groups of organic compounds including ketones, acids, and alcohols. Among these groups, ketones stood out because of their relative abundance and chemical diversity. One ketone (5-methyl-2-hexanone) was male-specific (figure 3) and eight others (2-hexanone, 3-methyl-2-pentanone, 4-heptanone, 2-heptanone, 6-methyl-2-heptanone, 3-octanone, 2-octanone, 2-nonanone) were, on average, more abundant in samples of males than of females (table 2). These data, coupled with findings that male brown rats produce a blend of seven ketones that attracts females [60], led us to hypothesize that some, or all, of the male deer mouse ketones may serve as sex-attractant semiochemicals.

We tested this hypothesis in field Exp. 2, which showed that traps baited with the ketone blend captured 3.4 times more females than unbaited control traps (figure 4), indicating that some or all ketones in this blend served as sex-attractant semiochemicals. Although it is conceivable that females responded to the ketone blend in recognition of ‘social signals’ that may mark a place for huddling together against the cold [19], breeding females usually occupy exclusive territories and nest solitarily [22,33]. Moreover, trap captures were female-biased irrespective of breeding season which occurs between March and October or even year-round under favourable conditions [21]. Drawing on a house mouse study [49], female deer mice may even avoid conspecific females. In house mice, traps baited with excreta-soiled bedding of females strongly deterred conspecific females (while attracting males), whereas traps baited with excreta-soiled bedding of males strongly attracted females [49], clearly indicating that excreta semiochemicals have a sexual communication function. Our interpretation that some or all ketones in the trap lure serve as sex-attractant semiochemicals would further be supported if these ketones were upregulated as male deer mice progress from juveniles to sexually mature adults, analogous to processes reported in male brown rats [60].

The strong female skew in deer mouse trap captures (figures 2, 4 and 5) could possibly be explained, if there were more females than males in populations, or if females were to be more likely than males to enter traps. Yet, the female:male capture ratio of mature or immature mice in control traps was 0:1 (Exp. 1), 2:2 (Exp. 2), and 1:0 and 6:9 (Exp. 3), suggesting that females were not more abundant or more likely to enter traps. Similarly, more female voles (9:2) and more female shrews (8:1) were captured in semiochemical-baited traps than in control traps, even though the female:male capture ratio in control traps was 2:2 (voles) and 1:2 (shrews).

Our data from Exp. 3 demonstrate that the sex steroid testosterone is a major sex-attractant semiochemical of male deer mice. When added to the ketone blend as a trap lure constituent, testosterone increased the blend’s attractiveness to female deer mice, while concurrently deterring immature males. Traps baited with lures comprising both the ketone blend and testosterone captured 8 times more mature females and 2.3 times more immature females than traps baited with the ketone blend alone (figure 5). Conversely, 9 out of 10 captured immature males avoided traps with testosterone as a trap lure constituent (figure 5). That immature females were captured in Exp. 3, but not in Exp. 2, is not likely due to contrasting ratios of mature and immature mice in local populations, because in both experiments traps were deployed in numerous locations. Rather, attraction and captures of immature females in Exp. 3 were likely due to the presence of testosterone as a lure constituent. In a previous field study, when wild house mice were offered a choice between paired traps that were baited with either the complete male sex-attractant semiochemical blend (including testosterone) or an incomplete blend (lacking testosterone), all 15 mature females and 18 out of 23 immature females were captured in traps baited with the semiochemical blend that included testosterone [12]. That the presence of sex steroid semiochemicals affects captures of immature mice was also evident in another paired-trap experiment. Traps baited with the female sex steroid semiochemicals progesterone and estradiol captured 8 mature male mice and 21 juvenile male mice, whereas corresponding unbaited control traps captured only a single juvenile male [12].

With captures of 40 deer mice in Exp. 3, the capture of only a single mature male is not likely a reflection of low population size. Rather, it may indicate that mature males sensed the ketone blend and testosterone in the surroundings of trap pairs and avoided the area which—naturally—may be an adaptive behaviour to avoid encounters with rival males. Following reports that house mice and brown rats respond to sex steroid semiochemicals (testosterone (males); progesterone, estradiol (females)) [12], deer mice are now the third rodent species shown to use testosterone as a sex-attractant signal. With testosterone being a ubiquitous hormone in the urine of all species of mammals, it is conceivable that testosterone serves as a sex-attractant signal not only in rodents but in diverse taxonomic orders. This inference is supported by our data showing that females of two distinct phylogenetic orders—vagrant shrews (Eulipotyphla) and field voles (Rodentia)—were significant by-captures in traps baited with excreta-soiled bedding containing testosterone (figure 2).

Our field data support the conclusion that the ketone blend contains essential sex-attractant semiochemicals produced by male deer mice. We selected these components based on their specificity and relatively greater abundance in urine/faeces odours of males than females (figure 2*a*,*b*). Conceivably, however, there is plasticity and redundancy in the ketone blend in that some components may be attractive on their own while others may be omittable without affecting the blend’s attractiveness. For example, 3-methyl-2-pentanone as a single representative of the ketone blend was sufficient to attract female deer mice in a trapping experiment with house mice [14]. Moreover, some components of the ketone blend may have the exclusive function of suppressing attraction of heterospecific rodents, while other ketones may have the dual function of attracting mates while deterring heterospecifics. These types of signal functions have been demonstrated in odour/pheromone blends of insects such as moths [61–63].

With ever-expanding knowledge about murine rodent sex pheromones or semiochemicals, opportunities arise to study semiochemical blends with respect to shared biosynthetic pathways, species-specificity of sex-attractant semiochemicals, and phylogenetic relatedness of species [3,63,64]. The presence of 2- and 4-heptanone in urine/faeces odours of male deer mice (figure 3) and male brown rats [60], and the presence of 3-methyl-2-pentanone in urine/faeces odours of male deer mice (figure 3) and male house mice [14], imply a shared biosynthetic pathway for these ketones. Pheromones or semiochemicals of similar structure, and shared biosynthetic pathways for pheromones and odourants, are well documented in closely related insect taxa [65] but—to our knowledge—have not yet been reported for mammals. The similarity of the ketone semiochemicals between male brown rats and male deer mice, and their dissimilarity with the male house mouse semiochemicals is surprising because the time-calibrated, family-level phylogenetic tree of rodents places the brown rat and the house mouse as sister species, and the deer mouse as a more distant relative [66]. With testosterone being a sexual communication signal of male brown rats, house mice and deer mice [12], there is distinct overlap in their signalling system. In male brown rats and house mice, specificity of the signalling system is achieved through the volatile sex-attractant components [3] that markedly differ between these two species. A similar type of mechanism may separate communication channels of deer mice and house mice and of deer mice and brown rats.

In conclusion, we show that the sex-attractant semiochemicals of male deer mice comprise some or all of nine volatile ketones and the less volatile sex steroid testosterone. As deer mice can be significant urban and agricultural pests [21], commercial development of this blend, or a less complex version thereof, as a trap lure seems warranted.

## Data Availability

All data are presented in the main body of the paper. Supplementary material is available online [67].

## References

[1] Howard WE, Cole RE. 1967 Olfaction in seed detection by deer mice. J. Mammal. **48**, 147–150. (10.2307/1378190)

[2] Howard WE, Marsh RE, Cole RE. 1968 Food detection by deer mice using olfactory rather than visual cues. Anim. Behav. **16**, 13–17. (10.1016/0003-3472(68)90100-0) 5639893

[3] Varner E, Jackson H, Mahal M, Takács S, Gries R, Gries G. 2020 Brown rats and house mice eavesdrop on each other’s volatile sex pheromone components. Sci. Rep. **10**, 17701. (10.1038/s41598-020-74820-4) 33077874 PMC7572391

[4] Sievert T, Laska M. 2016 Behavioral responses of CD-1 mice to six predator odor components. Chem. Senses **41**, 399–406. (10.1093/chemse/bjw015) 26892309

[5] Hurst JL. 1990 Urine marking in populations of wild house mice Mus domesticus Rutty. I. Communication between males. Anim. Behav. **40**, 209–222. (10.1016/S0003-3472(05)80916-9)

[6] Hurst JL. 1990 Urine marking in populations of wild house mice Mus domesticus Rutty. III. Communication between the sexes. Anim. Behav. **40**, 233–243. (10.1016/S0003-3472(05)80918-2)

[7] Hurst JL, Robertson D, Tolladay U, Beynon R. 1998 Proteins in urine scent marks of male house mice extend the longevity of olfactory signals. Anim. Behav. **55**, 1289–1297. (10.1006/anbe.1997.0650) 9632512

[8] Hurst JL, Payne CE, Nevison CM, Marie AD, Humphries RE, Robertson DHL, Cavaggioni A, Beynon RJ. 2001 Individual recognition in mice mediated by major urinary proteins. Nature **414**, 631–634. (10.1038/414631a) 11740558

[9] Leinders-Zufall T, Lane AP, Puche AC, Ma W, Novotny MV, Shipley MT, Zufall F. 2000 Ultrasensitive pheromone detection by mammalian vomeronasal neurons. Nature **405**, 792–796. (10.1038/35015572) 10866200

[10] Haga S *et al*. 2010 The male mouse pheromone ESP1 enhances female sexual receptive behaviour through a specific vomeronasal receptor. Nature **466**, 118–122. (10.1038/nature09142) 20596023

[11] Chamero P, Marton TF, Logan DW, Flanagan K, Cruz JR, Saghatelian A, Cravatt BF, Stowers L. 2007 Identification of protein pheromones that promote aggressive behaviour. Nature **450**, 899–902. (10.1038/nature05997) 18064011

[12] Takács S, Gries R, Gries G. 2017 Sex hormones function as sex attractant pheromones in house mice and brown rats. ChemBioChem **18**, 1391–1395. (10.1002/cbic.201700224) 28447367

[13] Novotny M, Harvey S, Jemiolo B, Alberts J. 1985 Synthetic pheromones that promote inter-male aggression in mice. Proc. Natl Acad. Sci. USA **82**, 2059–2061. (10.1073/pnas.82.7.2059) 3856883 PMC397491

[14] Varner E, Gries R, Gries G. 2021 Attractant blend compositions, devices and methods for attracting female mice. US patent no. US2022/0046917A1.

[15] Varner E, Gries R, Takács S, Fan S, Gries G. 2019 Identification and field testing of volatile components in the sex attractant pheromone blend of female house mice. J. Chem. Ecol. **45**, 18–27. (10.1007/s10886-018-1032-3) 30411204

[16] McLean BS, Barve N, Flenniken J, Guralnick RP. 2019 Evolution of litter size in North America’s most common small mammal: an informatics-based approach. J. Mammal. **100**, 365–381. (10.1093/jmammal/gyz057)

[17] Denys C, Taylor PJ, Aplin KP. 2017 The mammals of North America. In Handbook of the mammals of the world (eds DE Wilson, TE Lacher, RA Mittermeier). Barcelona, Spain: Lynx Edicions.

[18] Banfield AWF (ed). 1974 The mammals of Canada. Toronto, Canada: University of Toronto Press.

[19] Baker RH. 1983 Michigan mammals. Detroit, MI: Wayne State University.

[20] Nowak RM. 1999 Walker’s mammals of the world, vol. 2, 6th edn. Baltimore, MD: Johns Hopkins University Press.

[21] Witmer GW, Moulton RS. 2012 Deer mice (Peromyscus spp.) biology, damage, and management: a review. In Proc. 25th Vertebrate Pest Conf., vol. 25, pp. 213–219. Davis, CA: University of California.

[22] Wolff JO. 1989 Social behavior. In Advances in the study of Peromyscus (Rodentia) (eds G Kirkland, N Layne). Lubbock, TX: Texas Tech University Press.

[23] Fairbairn DJ. 1978 Dispersal of deer mice, Peromyscus maniculatus: proximal causes and effects on fitness. Oecologia **32**, 171–193. (10.1007/BF00366070) 28309396

[24] Dewsbury DA, Ferguson B, Hodges AW, Taylor SA. 1986 Tests of preferences of deer mice (Peromyscus maniculatus bairdi) for individuals and their odors as a function of sex and estrous condition. J. Comp. Psychol. **100**, 117–127. (10.1037/0735-7036.100.2.117)

[25] Birdsall DA, Nash D. 1973 Occurrence of successful multiple insemination of females in natural populations of deer mice (Peromyscus maniculatus). Evolution **27**, 106–110. (10.1111/j.1558-5646.1973.tb05922.x) 28563668

[26] Dewsbury DA. 1981 Social dominance, copulatory behavior, and differential reproduction in deer mice (Peromyscus maniculatus). J. Comp. Physiol. Psychol. **95**, 880–895. (10.1037/h0077842)

[27] Dewsbury DA. 1985 Interactions between males and their sperm during multi-male copulatory episodes of deer mice (Peromyscus maniculatus). Anim. Behav. **33**, 1266–1274. (10.1016/S0003-3472(85)80186-X)

[28] Ribble DO, Millar JS. 1996 The mating system of northern populations of Peromyscus maniculatus as revealed by radiotelemetry and DNA fingerprinting. Écoscience **3**, 423–428. (10.1080/11956860.1996.11682359)

[29] Kirkland GL, Layne JN. 1989 Advances in the study of Peromyscus (Rodentia). Lubbock, TX: Texas Tech University Press.

[30] Fisher HS, Hoekstra HE. 2010 Competition drives cooperation among closely related sperm of deer mice. Nature **463**, 801–803. (10.1038/nature08736) 20090679 PMC2824558

[31] Brown LE. 1969 Field experiments on the movements of Apodemus sylvaticus L. using trapping and tracking techniques. Oecologia **2**, 198–222. (10.1007/BF00379159) 28309326

[32] Selander RK. 1970 Behavior and genetic variation in natural populations. Am. Zool. **10**, 53–66. (10.1093/icb/10.1.53) 5437297

[33] Wolff JO. 1994 Reproductive success of solitarily and communally nesting white-footed mice and deer mice. Behav. Ecol. **5**, 206–209. (10.1093/beheco/5.2.206)

[34] Bendesky A, Kwon YM, Lassance JM, Lewarch CL, Yao S, Peterson BK, He MX, Dulac C, Hoekstra HE. 2017 The genetic basis of parental care evolution in monogamous mice. Nature **544**, 434–439. (10.1038/nature22074) 28424518 PMC5600873

[35] Khadraoui M, Merritt JR, Hoekstra HE, Bendesky A. 2022 Post-mating parental behavior trajectories differ across four species of deer mice. PLoS ONE **17**, e0276052. (10.1371/journal.pone.0276052) 36251655 PMC9576063

[36] Wolff JO, Cicirello DM. 1991 Comparative paternal and infanticidal behavior of sympatric white-footed mice (Peromyscus leucopus noveboracensis) and deermice (P. maniculatus nubiterrae). Behav. Ecol. **2**, 38–45. (10.1093/beheco/2.1.38)

[37] Gilbert BS, Cichowski DB, Talarico D, Krebs CJ. 1986 Summer activity patterns of three rodents in the Southwestern Yukon. Arct. Inst. N. Am. **39**, 204–207. (10.14430/arctic2075)

[38] Clarke JA. 1983 Moonlight’s influence on predator/prey interactions between short-eared owls (Asio flammeus) and deermice (Peromyscus maniculatus). Behav. Ecol. Sociobiol. **13**, 205–209. (10.1007/BF00299924)

[39] Davidson DL, Morris DW. 2001 Density-dependent foraging effort of deer mice (Peromyscus maniculatus). Funct. Ecol. **15**, 575–583. (10.1046/j.0269-8463.2001.00569.x)

[40] Bronson FH, Eleftheriou BE, Dezell HE. 1969 Strange male pregnancy block in deermice: prolactin and adrenocortical hormones. Biol. Reprod. **1**, 302–306. (10.1095/biolreprod1.3.302) 4331908

[41] Bronson FH, Marsden HM. 1964 Male-induced synchrony of estrus in deermice. Gen. Comp. Endocrinol. **4**, 634–637. (10.1016/0016-6480(64)90073-5) 14247601

[42] Bronson FH, Dezell HE. 1968 Studies on the estrus-inducing (pheromonal) action of male deermouse urine. Gen. Comp. Endocrinol. **1**, 339–343. (10.1016/0016-6480(68)90043-9) 5690661

[43] Teague LG, Bradley EL. 1978 The existence of a puberty accelerating pheromone in the urine of the male prairie deermouse (Peromyscus maniculatus bairdii). Biol. Reprod. **19**, 314–317. (10.1095/biolreprod19.2.314) 719090

[44] Lombardi JR, Whitsett JM. 1980 Effects of urine from conspecifics on sexual maturation in female prairie deermice, Peromyscus maniculatus bairdii. J. Mammal. **61**, 766–768. (10.2307/1380337) 7217792

[45] Knopf JL, Gallagher JF, Held WA. 1983 Differential, multihormonal regulation of the mouse major urinary protein gene family in the liver. Mol. Cell. Biol. **3**, 2232–2240. (10.1128/mcb.3.12.2232-2240.1983) 6656765 PMC370094

[46] Billitti JE, Lasley BL, Wilson BW. 1998 Development and validation of a fecal testosterone biomarker in Mus musculus and Peromyscus maniculatus. Biol. Reprod. **59**, 1023–1028. (10.1095/biolreprod59.5.1023) 9780305

[47] Ma W, Wiesler D, Novotny MV. 1999 Urinary volatile profiles of the deermouse (Peromyscus maniculatus) pertaining to gender and age. J. Chem. Ecol. **25**, 417–431. (10.1023/A:1020937400480)

[48] Jemiolo B, Alberts J, Sochinski-Wiggins S, Harvey S, Novotny M. 1985 Behavioural and endocrine responses of female mice to synthetic analogues of volatile compounds in male urine. Anim. Behav. **33**, 1114–1118. (10.1016/S0003-3472(85)80170-6)

[49] Musso AE, Gries R, Zhai H, Takács S, Gries G. 2017 Effect of male house mouse pheromone components on behavioral responses of mice in laboratory and field experiments. J. Chem. Ecol. **43**, 215–224. (10.1007/s10886-017-0819-y) 28130740

[50] Cavaliere RM, Silvotti L, Percudani R, Tirindelli R. 2020 Female mouse tears contain an anti-aggression pheromone. Sci. Rep. **10**, 2510. (10.1038/s41598-020-59293-9) 32054888 PMC7018997

[51] Karlson P, Lüscher M. 1959 ‘Pheromones’: a new term for a class of biologically active substances. Nature **183**, 55–56. (10.1038/183055a0) 13622694

[52] Wyatt TD. 2017 Pheromones. Curr. Biol. **27**, R739–R743. (10.1016/j.cub.2017.06.039) 28787598

[53] Takács S, Musso AE, Gries R, Rozenberg E, Borden JH, Brodie B, Gries G. 2017 New food baits for trapping house mice, black rats and brown rats. Appl. Anim. Behav. Sci. **200**, 130–135. (10.1016/j.applanim.2017.11.011)

[54] Schneider JE, Wysocki CJ, Nyby J, Whitney G. 1978 Determining the sex of neonatal mice (Mus musculus). Behav. Res. Methods Instrum. **10**, 105–105. (10.3758/BF03205110)

[55] Montoto LG, Arregui L, Sánchez NM, Gomendio M, Roldan ERS. 2012 Postnatal testicular development in mouse species with different levels of sperm competition. Reproduction **143**, 333–346. (10.1530/REP-11-0245) 22187670

[56] Safranski TJ, Lamberson WR, Keisler DH. 1993 Correlations among three measures of puberty in mice and relationships with estradiol concentration and ovulation. Biol. Reprod. **48**, 669–673. (10.1095/biolreprod48.3.669) 8452942

[57] van den Dool H, Kratz P. 1963 A generalization of the retention index system including linear temperature programmed gas–liquid partition chromatography. J. Chromatogr. A **11**, 463–471. (10.1016/S0021-9673(01)80947-X) 14062605

[58] R Core Team. 2022 R: a language and environment for statistical computing. Vienna, Austria: R Foundation for Statistical Computing. See https://www.r-project.org/.

[59] Hoffmann F, Musolf K, Penn DJ. 2009 Freezing urine reduces its efficacy for eliciting ultrasonic vocalizations from male mice. Physiol. Behav. **96**, 602–605. (10.1016/j.physbeh.2008.12.014) 19150619

[60] Takács S, Gries R, Zhai H, Gries G. 2016 The sex attractant pheromone of male brown rats: identification and field experiment. Angew. Chem. Int. Ed. **55**, 6062–6066. (10.1002/anie.201511864) 27060700

[61] Gries G, Gries R, Khaskin G, Slessor KN, Grant GG, Liška J, Kapitola P. 1996 Specificity of nun and gypsy moth sexual communication through multiple-component pheromone blends. Naturwissenschaften **83**, 382–385. (10.1007/BF01142006)

[62] Grant GG, Langevin D, Liška J, Kapitola P, Chong JM. 1996 Olefin inhibitor of gypsy moth, Lymantria dispar, is a synergistic pheromone component of nun moth, L. monacha. Naturwissenschaften **83**, 328–330. (10.1007/BF01152217)

[63] Junker RR *et al*. 2018 Covariation and phenotypic integration in chemical communication displays: biosynthetic constraints and eco-evolutionary implications. New Phytol. **220**, 739–749. (10.1111/nph.14505) 28256726

[64] Symonds MRE, Elgar MA. 2008 The evolution of pheromone diversity. Trends Ecol. Evol. **23**, 220–228. (10.1016/j.tree.2007.11.009) 18308422

[65] Tillman JA, Seybold SJ, Jurenka RA, Blomquist GJ. 1999 Insect pheromones: an overview of biosynthesis and endocrine regulation. Insect Biochem. Mol. Biol. **29**, 481–514. (10.1016/s0965-1748(99)00016-8) 10406089

[66] Swanson MT, Oliveros CH, Esselstyn JA. 2019 A phylogenomic rodent tree reveals the repeated evolution of masseter architectures. Proc. R. Soc. B **286**, 20190672. (10.1098/rspb.2019.0672) PMC6532498 31064307

[67] Varner E, Gries R, Takacs S, Jackson H, Purdey L, Gofredo D *et al*. 2024. Supplementary material from: Identification and field testing of sex-attractant semiochemicals produced by male deer mice, Peromyscus maniculatus. FigShare (10.6084/m9.figshare.c.7576671) PMC11651908 39698158

